# The Role of Lymphostatin/EHEC Factor for Adherence-1 in the Pathogenesis of Gram Negative Infection

**DOI:** 10.3390/toxins2050954

**Published:** 2010-05-05

**Authors:** Jan-Michael A. Klapproth

**Affiliations:** Division of Digestive Diseases, Emory University, Atlanta GA 30322, USA; Email: jklappr@emory.edu; Tel.: +1-404-727-5638

**Keywords:** lymphocyte inhibitory factor A/EHEC factor for adherence-1, lymphostatin, Gram negative bacterial toxin, Enteropathogenic *E. coli*, Enterohemorrhagic *E. coli*, immunosuppression, *Citrobacter rodentium*, intestinal epithelial barrier function

## Abstract

Lymphostatin/EHEC factor for adherence-1 is a novel large toxin represented in various Gram negative bacteria, highly associated with the development of infectious diarrhea and hemolytic uremic syndrome. *In vitro* and *in vivo* experiments identified lymphostatin/EFA-1 as a toxin with a central role in the pathogenesis of Gram negative bacteria, responsible for bacterial adhesion, intestinal colonization, immunosuppression, and disruption of gut epithelial barrier function.

## 1. Introduction

Gastrointestinal infections and diarrhea are a worldwide problem with more than 1 billion cases and approximately 2 million deaths annually [1]; 80% of which children aged 6 months to 3 years. Infectious diarrhea caused by Gram negative bacteria can be due to Enteropathogenic *E. coli* (EPEC) in developing countries [2], and Enterohemorrhagic *E. coli* (EHEC) in developed nations [3], complicated by dehydration, weight loss, and hemolytic-uremic syndrome (HUS) [4]. 

Pathogenic *E. coli* harbor a large arsenal of pathogenicity genes, located on plasmids and the bacterial chromosome, and effector proteins. An example is the EAF-plasmid (EPEC adherence factor) [5] encoding for a battery of adhesion factors. An additional example is the “locus of enterocytes effacement” (LEE) [6] pathogenicity island located on the bacterial chromosome, encoding for more than 40 proteins necessary for the type III secretion system and effector proteins that modify and neutralize host cellular functions. However, not all genes encoding for effector proteins and associated factors implicated in Gram negative pathogenesis are located on the LEE island, and one such example is *lifA/e-1* (lymphocyte inhibitory factor A/EHEC factor for adherence-1). Recently, it has become clear that *lifA/efa-1* assumes a central role in the pathogenesis of infectious diarrhea.

## 2. Molecular Studies

Pathogenic *E. coli*, like EPEC and EHEC, cause diarrheal diseases in all age groups. EPEC strains predominantly colonize the small intestine and rely on the virulence plasmid-encoded bundle-forming pili for the formation of microcolonies (localized adherence) and LEE-encoded intimin for intimate attachment to epithelial cells and effacement of micovilli [7]. In contrast, EHEC preferentially colonizes the distal ileum and colon with tropism for follicle-associated epithelium [8]. Like EPEC, EHEC harbors the LEE pathogenicity island, but also possesses phage-encoded Shiga toxins 1 and 2 that induce an intense inflammatory response [9]. 

*lifA*/*efa-1* is found in pathogenic, but not non-pathogenic, *E. coli* strains, and is detected exclusively in strains that are capable of attaching and effacing activity, a phenotype encoded by the LEE [10,11]. This gene is present in 87.5% in EPEC, including atypical EPEC that do not bear the bfp-plasmid [10,12,13], 89.7%-100% of EHEC, in particular serotypes O5, O26, O23, and O111 [11,14,15,16,17], rabbit Enteropathogenic *E. coli* (REPEC) serotypes 89/39 and 84/110-1 [18,19,20], 100% of *Hafnia alvei*, and mouse pathogen *Citrobacter rodentium*. *lifA*/*efa-1* is located on the bacterial chromosome in EPEC, REPEC, and *C. rodentium* [21]. A similar, large gene with 3,169 codons encoding for a putative 362 kDa product was identified on the large 93 kb to 104 kb pO157 virulence plasmid in EHEC strain O157:H7, termed *toxB/L7095* [22]. Over its entire length, nucleotide and residue similarity of *toxB* in comparison to *lifA/efa-1* was only 28% and 47%, respectively. However, like *lifA/efa-1* and large Clostridial cytotoxins, the N-terminus contained sequences encoding for a glucosyltransferase. A truncated *lifA/efa-1* pseudogene (*efa-1’*) is located in the O island 122 at *z4332* and *z4333* on bacterial chromosome of serotype O157:H7 [23]. 

Interestingly, *lifA*/*efa-1* is also present in various *Chlamydia* spp strains. *C. muridarium* harbors three, *C. psittaci* a single *lifA*/*efa-1* gene, *C. trachomaits* a shortened pseudogene, whereas it is not represented in *C. pneumonia* [24]. Therefore, it is conceivable that this toxin is responsible for organ specificity of different *Chlamyida* spp strains [25]. 

The association of *lifA*/*efa-1* with the pathogenesis of infectious diarrhea was established through molecular studies of stool samples [26]. In contrast to typical EPEC strains, atypical EPEC lack the 69 kb pathogenicity EAF-plasmid and express less bacterial effector proteins. DNA microarray analysis of 182 virulence genes identified *lifA*/*efa-1* as the gene with the strongest statistical association with infectious diarrhea caused by atypical EPEC strains among Norwegian children, recently confirmed in a patient population from Brazil presenting with acute diarrhea [27].

Several groups have shown that *lifA*/*efa-1* in EHEC and atypical EPEC strains can be part of larger novel gene cluster (O122, I_RW1374_, OI-122) [28], responsible for increased pathogenicity. The 29 kb pathogenicity complex OI-122 consists of 26 genes, is divided into three modules, and is represented in EHEC O157:H- with a variable repertoire in non-O157:H7 serotypes. Putative virulence genes are found in module 1, constituting genes *Z4318*, *pagC*, and *Z4322*. Module 2 is composed of *Z4323*, *ent*, *nleB*, and *nleB*, whereas the third module is made up by *lifA*/*efa-1* alone [29]. Each of these modules is significantly associated with the development of HUS in EHEC O157:H-, but only module 3 is involved in non-O157 Shiga toxin-producing *E. coli* (STEC). Especially *pagC*, *Z4322*, *ent*, *nleE*, *nleB*, and *lifA/efa-1* appear to be more prevalent in HUS caused by non-O157 STEC [29,30]. Further, preliminary phenotypic characterization identified *ent*, *nleB*, and *lifA/efa-1* to be important for bacterial colonization, and *pagC*, *ent*, *nleB*, and *lifA/efa-1* as factors responsible for increased virulence. Genes implicated in survival of infected mice include *ent* and *nleB*, with *nleB* possibly modulating the infectious dose of non-O157 STEC to humans, thereby contributing to the low infectious dose of this Gram negative pathogen. Given the association of *nleB* and *lifA/efa-1* with the development of HUS, testing for the presence of both genes has been proposed for clinical identification in addition to other methods. From these studies it has become clear that lymphostatin/EFA-1 does not act alone. Interestingly, even cases of HUS caused by atypical EPEC are strictly dependent on the presence of *lifA*/*efa-1* [14]. 

Besides *lifA/efa-1*, *nleB* has also been characterized further regarding its biological activity. It was shown that *nleB* augments the inhibitory activity of *nleE* on Nf-kB through IkB stabilization, preventing its degradation [31]. In particular, *nleE* is required for maximal inhibition of TNF-α induced IL-8 expression *in vitro*, pointing to a possible immunosuppressive function of these two bacterial effector proteins.

**Figure 1:**
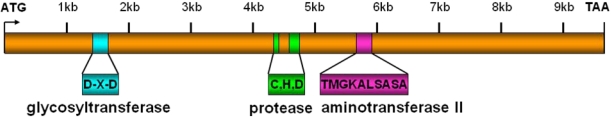
*lifA/efa-1* encodes for lymphostatin/EFA-1, a toxin present in EPEC, EHEC, *C. rodentium* and Chlamydiae strains. *lifA/efa-1* consists of approximately 9.6 kb with a C/G content of 42% and putative protein molecular weight of 360 kDa. DNA sequencing analysis confirmed significant similarity among various Gram negative bacteria. *lifA/efa-1* encodes for three enzymatic activities: a glycosyltransferase - (D-X-D, 1.6 kb), protease-(C, H, D, 4.5, 4.8 kb), and aminotransferase motif (TMGKALSASA, 5.8 kb).

*lifA*/*efa-1* encodes for a toxin with a calculated size of approximately 360 kDa (Figure 1), one of the largest known proteins in Gram negative bacteria [10]. Sequencing analysis identified three critical motifs also present in other pathogenic bacteria: glucosyltransferase-, protease-, and aminotransferase motif. The glucosyltransferase motif consists of two critical aspartic acid residues separated by a single codon (Figure 1; DXD), and its activity is essential in the pathogenesis of large Clostridial cytotoxins, inhibiting small GTPases like Ras, Rac, Rho, and Cdc42 through glycosylation [32]. The N-terminal putative products of *lifA/efa-1* exhibits 38% amino acid similarity to *Clostridium difficile* toxin A and B [16]. The second activity associated with the pathogenesis of Gram negative bacteria is the protease motif, catalyzing proteolytic cleavage of the C-terminal fatty acid modification of Rho [33]. The three critical residues for the protease activity are a cysteine, histidine, and aspartic acid (Figure 1; C,H,D). No role in the pathogenicity of Gram negative bacteria has been identified for the aminotransferase motif, consisting of ten residues (Figure 1, TMGKALSASA).

## 3. Functions of Lymphostatin/EFA-1

Initially, lymphostatin/EFA-1 was characterized as an immunosuppressive bacterial effector protein. *In vitro* studies with T lymphocytes isolated from peripheral blood [34], gastrointestinal mucosa [35], and spleen [36] have shown that lymphostatin/EFA-1 inhibits mitogen-activated cytokine expression. Pre-incubation of lymphocyte cultures with lysates from EPEC, EHEC, and *C. rodentium* and subsequent T cell receptor stimulation lead to a dose dependent inhibition of IL-2, IL-4, and IFN-γ transcription and protein expression, and proliferation [34,35,37]. This immunosuppressive effect is independent of secondary effects, like apoptosis, IL-10, TGF-β, or the presence of macrophages [36]. The immunosuppressive function is present in the bacterial supernatant with 20-fold increased inhibitory activity in comparison to bacterial cell lysates [35]. 

At this time it can only be speculated about the immunosuppressive function of lymphostatin in Chlamydia strains. Chlamydiae are obligatory intracellular pathogenic bacteria that are strictly dependent on tryptophan. Host synthesis of tryptophan is negatively regulated by IFN-γ. IFN-γ induces indolamine-2,3-dioxygenase and converts L-tryptophan into L-formylkynurenin [38]. Therefore, it is conceivable that lymphostatin/EFA-1 from Chlamydia strains suppresses mucosal IFN-γ expression, allowing intracellular persistence [24]. 

As mentioned above, *C. trachomatis* encodes for a *lifA*/*efa-1* pseudogene with significant homology to *C. difficile* toxin A and B. Previously, it has been shown that the N-terminal glucosyltransferase activity in *C. trachomatis* induces cellular ballooning, disassembly of actin fibers, and collapse of cytoskeleton in epithelial cells which is morphologically identical to changes induced by Clostridial toxins A and B [25].

In addition, our group has recently shown that lymphostatin/EFA-1 regulates intestinal epithelial barrier function *in vitro* and *in vivo* [39]. Infection of polarized enteric epithelial cells with *C. rodentium* led to inhibition of small GTPase Cdc42 and activation of RhoA. Inhibition of Cdc42 and activation of RhoA in Caco-2 cells were reversed in cultures incubated with *C. rodentiumlifA*/*efa-1* glucosyltransferase and protease motif mutant strains, respectively (Figure 1, above). Inactivation of Cdc42 induced disassembly of adherens junctions, whereas stimulation of RhoA disrupted tight junctions in intestinal epithelial cell cultures. 

Besides an effect on the immune system and intestinal barrier function, lymphostatin/EFA-1 appears to function as an adhesion factor for EHEC and EPEC strains *in vitro* [16,18], hence the name. In EAF-negative EPEC, deletion of chromosomal *lifA*/*efa-1* and pre-incubation with neutralizing antibodies against lymphostatin/EFA-1 decreased bacterial adhesion to epithelial cells. Similar experiments with EHEC showed that successive inactivation of EHEC *lifA*/*efa-1* on the 93 kb pathogenicity plasmid and chromosomal pseudogene lead to a 4- and 12-fold reduction in adhesion to epithelial cells *in vitro*, respectively [40]. However, *in vivo* experiments with cattle and sheep assigned lymphostatin/EFA-1 only a minor role in intestinal colonization. In comparison to wild type, mutation of *lifA*/*efa-1* gene or pseudogene in EHEC O157:H7 had no effect on the number of adhering bacteria in the intestines of infected animals. Further, a genome-wide search for EHEC adhesion molecules by transposon mutagenesis identified 59 bacterial genes that play a role in colonization of cattle intestine, *lifA*/*efa-1* was not among them [41]. This is in sharp contrast to experiments with calves [40] and mice [21] as both, *lifA*/*efa-1* glucosyltransferase and protease motifs were essential for successful intestinal colonization. However, colonization studies with *lifA/efa-1* wild type and mutant *C. rodentium* in mice utilized strains with a 5’ located stop codon and weak 3’ ribosomal binding site and start codon in the insertion sequence. It is possible that expression of genes located downstream of the mutations is compromised and therefore a clear role in colonization of mouse intestine cannot be assigned for either motif.

In addition to an effect on eukaryotic cellular functions, studies have shown that lymphostatin/EFA-1 positively regulates endogenous prokaryotic protein expression for genes located on the LEE pathogenicity island [22,42]. Mutations of *lifA*/*efa-1* gene and pseudogene lead to an 8-fold reduction in bacterial *espD* expression, a protein critical for the type III secretion system. Similarly, inactivation of *toxB* decreased production and secretion of adhesion molecules EspA, EspB, and Translocated intimin receptor, as well as the number of bacterial microcolonies on intestinal epithelial cells. Therefore, it is conceivable that lymphostatin/EFA-1 and ToxB directly and indirectly regulate bacterial effector proteins, in particular, those involved in bacterial adhesion. However, these findings have not been verified in other Gram negative pathogens, like EPEC or RDEC strains. 

Like tripartite toxin A and B from *Clostridium* spp, lymphostatin/EFA-1 harbors an N-terminal glucosyltransferase motif, when expressed alone, does not glycosylate small GTPases RhoA and Rab *in vitro* with UDP-glucose as a cofactor. However, it is possible that lymphostatin/EFA-1 targets a single or multiple different GTPases glycosylating the preserved threonine, utilizing a UDP-hexose like UDP-N-acetylglucosamine or UDP-galactose.

## 4. Conclusion and Future Outlook

Lymphostatin/EFA-1 is a large multi-functional toxin encoded by *lifA/efa-1* and expressed in various Gram negative pathogenic bacteria. *lifA/efa-1* is significantly associated with the development of diarrhea and systemic complications caused by enteric pathogens, including EPEC and EHEC strains. Investigations of specific motifs implicated in the suppression of cytokine expression and regulation of bacterial adhesion molecules will clarify the role of lymphostatin/EFA-1 in the pathogenesis of Gram negative bacteria. Like large Clostridial cytotoxins, it is of interest to identify additional intracellular pathways affected by this toxin, in particular, those involving small GTPases. Further, there is limited knowledge about secretion and the cellular ligand of lymphostatin/EFA-1, whether this toxin is cleaved into smaller subunits, and the structure of this protein which is in part explained by its toxicity when expressed as a recombinant protein. It remains to be determined whether lymphostatin/EFA-1 is an ideal candidate protein for vaccine development to prevent devastating sequelae of enteric infections.
